# Time-varying whole-brain functional network connectivity coupled to task engagement

**DOI:** 10.1162/netn_a_00051

**Published:** 2018-10-01

**Authors:** Hua Xie, Javier Gonzalez-Castillo, Daniel A. Handwerker, Peter A. Bandettini, Vince D. Calhoun, Gang Chen, Eswar Damaraju, Xiangyu Liu, Sunanda Mitra

**Affiliations:** Department of Electrical and Computer Engineering, Texas Tech University, Lubbock, TX, USA; Section on Functional Imaging Methods, National Institute of Mental Health, National Institutes of Health, Bethesda, MD, USA; Section on Functional Imaging Methods, National Institute of Mental Health, National Institutes of Health, Bethesda, MD, USA; Section on Functional Imaging Methods, National Institute of Mental Health, National Institutes of Health, Bethesda, MD, USA; Section on Functional Imaging Methods, National Institute of Mental Health, National Institutes of Health, Bethesda, MD, USA; Functional MRI Facility, National Institute of Mental Health, National Institutes of Health, Bethesda, MD, USA; The Mind Research Network, Albuquerque, NM, USA; Department of Electrical and Computer Engineering, University of New Mexico, Albuquerque, NM, USA; Scientific and Statistical Computing Core, National Institute of Mental Health, National Institutes of Health, Bethesda, MD, USA; The Mind Research Network, Albuquerque, NM, USA; Department of Electrical and Computer Engineering, University of New Mexico, Albuquerque, NM, USA; Department of Electrical and Computer Engineering, Texas Tech University, Lubbock, TX, USA; Department of Electrical and Computer Engineering, Texas Tech University, Lubbock, TX, USA

**Keywords:** Whole-brain connectivity pattern, Cognitive marker, Task-evoked connectivity dynamics, Cognitive dynamics, Brainwide integration

## Abstract

Brain functional connectivity (FC), as measured by blood oxygenation level-dependent (BOLD) signal, fluctuates at the scale of 10s of seconds. It has recently been found that whole-brain dynamic FC (dFC) patterns contain sufficient information to permit identification of ongoing tasks. Here, we hypothesize that dFC patterns carry fine-grained information that allows for tracking short-term task engagement levels (i.e., 10s of seconds long). To test this hypothesis, 25 subjects were scanned continuously for 25 min while they performed and transitioned between four different tasks: working memory, visual attention, math, and rest. First, we estimated dFC patterns by using a sliding window approach. Next, we extracted two engagement-specific FC patterns representing active engagement and passive engagement by using *k*-means clustering. Then, we derived three metrics from whole-brain dFC patterns to track engagement level, that is, dissimilarity between dFC patterns and engagement-specific FC patterns, and the level of brainwide integration level. Finally, those engagement markers were evaluated against windowed task performance by using a linear mixed effects model. Significant relationships were observed between abovementioned metrics and windowed task performance for the working memory task only. These findings partially confirm our hypothesis and underscore the potential of whole-brain dFC to track short-term task engagement levels.

## Author Summary

In this study, we hypothesized that whole-brain dynamic functional connectivity (FC) patterns carry fine-grained information that allows for tracking short-term task engagement levels. We derived three task engagement markers from whole-brain dynamic FC pattern, that is, dissimilarity between dynamic FC patterns and high/low-engagement FC patterns, as well as brainwide integration level. We employed a linear mixed effects model to relate those task engagement markers with short-term task performance, and confirmed our hypothesis with the working memory task.

## INTRODUCTION

Functional connectivity (FC) analyses of resting-state functional magnetic resonance imaging (fMRI) data have consistently revealed sets of spatially distributed and temporally correlated brain regions, which correspond to canonical functions such as vision, audition, language, memory, and attention (Smith et al., 2009). Spontaneous fluctuations of FC during rest over short timescales (e.g., seconds to minutes), commonly referred to as FC dynamics (Hutchison et al., 2013), are believed to be primarily driven by neuronal phenomena, as evidenced by studies using simultaneous fMRI and electrophysiological recordings (Chang et al., 2013). Moreover, mounting evidence emphasizes the potential biological and cognitive significance of blood oxygenation level-dependent (BOLD) fMRI FC dynamics evaluated on the brain as a whole (e.g., considering all possible region-to-region connections). Along those lines, Allen et al. (2014) proposed a pipeline to investigate whole-brain dynamic FC (dFC) during rest, also called dynamic functional network connectivity (dFNC). The pipeline studies the time-varying connectivity between pairs of timecourses coming from independent networks/components, using a combination of spatial independent component analysis (ICA), and *k*-means clustering of sliding window correlation matrices. The identified FC states were suggested to reflect shifts in ongoing cognition during rest. This approach has recently been shown to be highly replicable (Abrol et al., 2017), predictive of mental illness (Rashid et al., 2016), and correlate with multimodal imaging modalities (Allen, Eichele, Wu, & Calhoun, 2013).

Subsequently, others have studied task modulation of FC patterns. Shirer and colleagues (2012) reported that subject-driven cognitive states (i.e., episodic memory, music, subtraction, and rest) could be correctly classified using whole-brain FC patterns estimated with a window length (WL) as short as 30–60 s. Similarly high clustering accuracy has been achieved by using *k*-means to segment task-evoked dFC patterns to identify the underlying cognitive task at both individual (Gonzalez-Castillo et al., 2015) and group levels (Xie et al., 2017). Nevertheless, it remains an open question whether and how task-evoked FC dynamics can be used beyond task identification. More specifically, we are interested to investigate whether spontaneous FC fluctuations during the performance of the task can be related to behavioral fluctuations.

Initial evidence suggests that task-evoked FC dynamics accompanying demanding tasks may indeed carry such detailed information (Gonzalez-Castillo & Bandettini, 2017). Yet, how to extract behaviorally relevant features from whole-brain dFC patterns remains a matter of debate. For example, Shine et al. (2016) focused on the level of brainwide information integration during an N-back working memory task, and reported that more integrated FC configurations, as reflected by a higher between-module connectivity, were associated with better task performance. Somehow contradictorily, Sadaghiani and colleagues (2015) found that it was a more modular—and therefore less integrated—network structure that led to improved perceptual efficiency for a continuous auditory detection task. Given the discrepancy, we are interested to evaluate how brain network structure reorganizes according to short-term task engagement level from a graph-theoretic perspective.

Additionally, an alternative approach would be to compute representative whole-brain FC patterns that describe how the brain is functionally organized during periods of high and low engagement (HE/LE) during a given task, and use some measure of dissimilarity between windowed dFCs and those HE/LE representative patterns to track short-term engagement level indexed by task performance. Here we make several claims, which lead to the following testable hypothesis.

**Claim 1:** Short-term task engagement levels, that is, the amount of mental effort dedicated to the task, are encoded in whole-brain FC dynamics.
**Claim 2:** The resting whole-brain FC pattern can be regarded as a non-task-specific marker of low engagement/passive engagement during task performance. Such a low-engagement FC (LE-FC) representative pattern is obtained here as the *k*-means centroid associated with resting periods, which serves as a prototype FC pattern for rest.
**Claim 3:** Task-related whole-brain FC patterns can be regarded as task-specific markers of high engagement/active engagement with a given task. Such task-specific high-engagement FC (HE-FC) representative patterns are estimated here using *k*-means centroids for active tasks.
**Hypothesis:** The distance between windowed dFCs and HE-FC/LE-FC patterns reflects ongoing changes in the task engagement levels, as reflected in variations in short-term task performance (e.g., faster/more accurate responses). dFC during a given window is expected to indicate better performance if it is more similar to the HE-FC pattern, while higher similarity to the LE-FC pattern may indicate worse performance.

**Figure 1.**
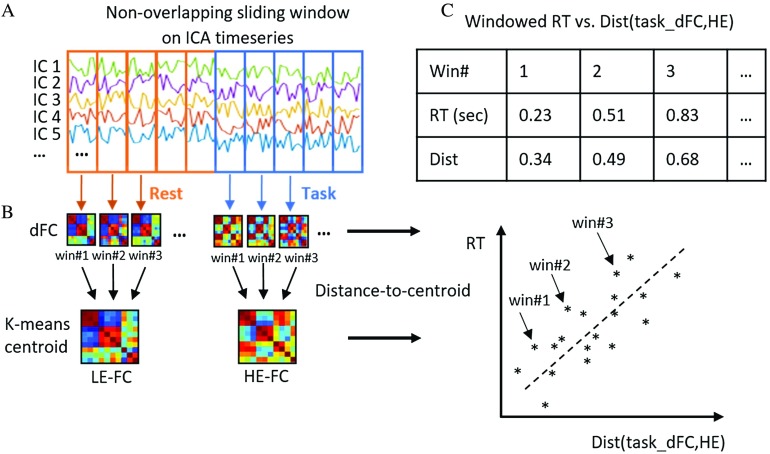
Schematic of the analysis pipeline. (A) dFC patterns were first computed using the windowed time series, obtained via group ICA, as input. (B) Unsupervised *k*-means clustering was then applied on the vectorized dFCs to obtain representative HE-FC or LE-FC patterns. (C) Distance between task-dFCs and the corresponding task-specific HE-FC pattern defined as *dist*(*task_dFC*, *HE*) are plotted against RT. A significant positive relationship between the two variables should be observed if our hypothesis holds.

To test this hypothesis, we used a continuous multitask dataset, part of which was published in (Gonzalez-Castillo et al., 2015) to test a different hypothesis, and also incorporated additional data with the same experimental design collected as the follow-up. We tested three task engagement markers derived from whole-brain dFC patterns, that is, brainwide integration level as well as distance between dFC patterns and HE/LE-FC patterns. We first estimated dFC from ICA time courses in terms of windowed whole-brain functional network connectivity patterns (WL = 45 s). Then, we estimated the temporal evolution of global brain integration levels as indexed by each dFC’s participation coefficient. We applied *k*-means algorithm to extract representative HE/LE-FC patterns as defined above. Cluster labels were assigned based on the experimental paradigm, and distance-to-centroid metrics were computed for all available windowed connectivity estimates. Finally, we evaluated if those three engagement markers correlated well with windowed behavioral measures of task engagement (e.g., windowed reaction time, RT). See Figure 1 for a schematic of the analysis pipeline to compute distance-to-centroid metrics.

We performed these analyses separately on the three available active tasks: 2-back working memory (memory), mental calculation (math), and visual attention (video). In this exploratory work, we were only able to partially confirm our hypothesis for one of the three tasks available, namely the 2-back memory task. As we shall discuss, the negative findings for the math and video task are probably due to task engagement not being the primary variance contributor to the behavioral metrics available for these two tasks.

## RESULTS

### Clustering Accuracy Versus Behavior

The average response accuracy, missing rate and RT within each window calculated following the previously used procedures (Gonzalez-Castillo et al., 2015), are reported in Table 1, which shows overall high task compliance across three active tasks.

**Table 1.** Average and standard deviation of RT, response accuracy, and missing rate

	**Memory**	**Math**	**Video**
RT (s)	1.00 ± 0.37	2.27 ± 0.35	1.34 ± 0.18
Accuracy (%)	93.30 ± 5.55	94.39 ± 4.98	66.63 ± 16.38
Missing (%)	13.23 ± 14.78	1.53 ± 2.54	30.50 ± 15.08

The average clustering accuracy describing the overall agreement between *k*-means partitions and ground truth task engagement across all 24 participants is 78.52%, suggesting in general the *k*-means algorithm could successfully group dFC patterns according to ongoing tasks despite the algorithm not being provided with any information about task timing.

**Figure 2.**
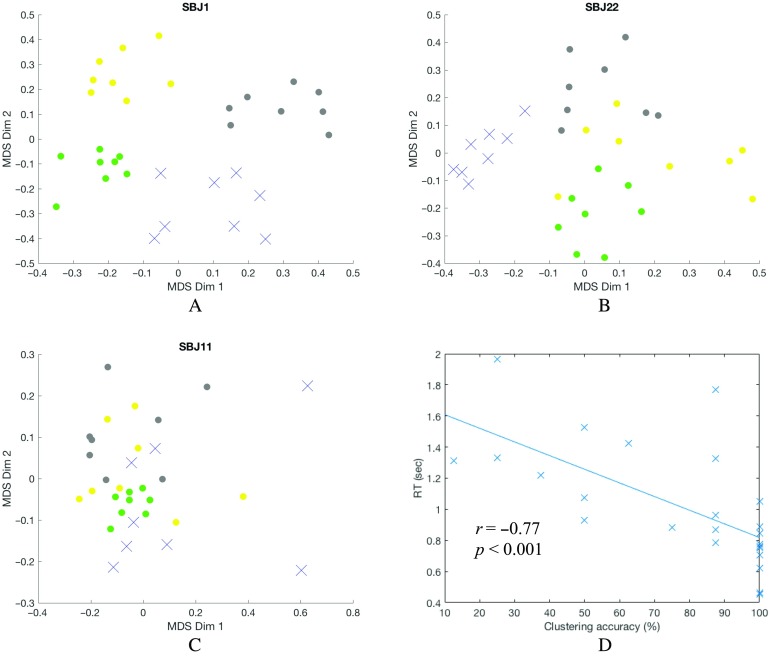
Multidimensional scaling (MDS) 2D projection of dFCs from three subjects with different overall task performance (A, B, and C), and clustering accuracy vs. RT for each subject during the working memory task (D). The dFCs are color coded based on the task. Rest: gray dot; memory: blue crosshair; video: yellow dot; math: green dot. (A) Subject 1 is a good subject with well distinguishable dFNC structure leading to very high overall clustering accuracy (100%). (B) Subject 22 is a mediocre performer with a few outliers leading to relatively high overall clustering accuracy (84.38%). (C) Subject 11 is a bad performer, and the lack of structure led to degraded overall clustering accuracy (53.12%). (D) Clustering accuracy was correlated with average RT for the memory task. Each cross-represented a subject.

To gain intuition of how clustering relates to behavior, we now focus on three representative subjects (SBJ 1, 22, and 11) with different performance levels (e.g., good, medium, and bad performance). Figure 2A shows a 2D visualization of dFCs and their cluster assignments (as colors) for a subject with overall good task performance obtained with multidimensional scaling (MDS). Similarly, Figure 2B shows the same result for a representative subject with mediocre performance and Figure 2C for one with bad overall task performance. For subjects with good performance (Figure 2A), dFCs appear to be highly organized according to the ongoing task, so that dFCs associated with a given task (as indicated by color) cluster together, and separate from those associated with the other tasks. In fact, for such a subject, *k*-means produces 100% clustering accuracy. As for a mediocre performer shown in Figure 2B, the equivalent 2D projection shows how the general 4-group structure is mostly preserved (yet with a few outliers present), leading to a clustering accuracy of 84.38%. For bad performers (Figure 2C), the 2D projection of dFCs becomes much less structured, reflecting weaker task modulation and leading to poor *k*-means clustering accuracy (53.12% for the subject depicted in Figure 2C).

We observed that clustering accuracy correlated with overall task performance across all tasks as previously reported (Gonzalez-Castillo et al., 2015). We next tested if that would be the case on a task-by-task basis. We found that the memory clustering accuracy was significantly correlated with subject’s average task performance metrics: RT (*r* = −0.77; *p*_*FDR*_ < 0.001; *df* = 22, shown in Figure 2D), missing rate (*r* = −0.62; *p*_*FDR*_ < 0.001; *df* = 22), and response accuracy (*r* = 0.43; *p*_*FDR*_ = 0.019; *df* = 22) using Spearman correlation (see also Figure 2A–C). All *p* values have been false discovery rate (FDR) corrected for multiple comparisons. We failed to find an equivalent significant relationship between clustering accuracy and average behavioral metrics for the other two active tasks.

### Tracking Short-Term Engagement Level

Clustering accuracy provides a limited picture of the behavioral significance of the clustering structure. To further understand the behavioral interpretation of whole-brain dFC, we evaluated three candidate metrics derived from whole-brain dFC, that is, *dist*(*task_dFC*, *HE*), *dist*(*task_dFC*, *LE*), and mean participation coefficient (*B*_*T*_). *dist*(*task_dFC*, *HE*) and *dist*(*task_dFC*, *LE*) refer to the distance of each windowed dFCs to its corresponding high-/low-engagement FC pattern (termed as HE-FC and LE-FC respectively) for a given subject and task. These distance measures reflect the overall dissimilarity between each dFC and engagement-specific FC patterns. Besides, we also computed the mean participation coefficient (*B*_*T*_) for each dFC by using brain connectivity toolbox (Rubinov & Sporns, 2010; https://sites.google.com/site/bctnet/), which quantifies the degree that a given brain region connects across different modules/networks. An overall higher *B*_*T*_ represents higher between-module connectivity, hence marks a more globally integrated brain state. As previously mentioned, task performance was evaluated in terms of window-averaged RT, response accuracy, and missing rate. Our goal is to determine the relationship between before-mentioned dFC-derived metrics and task performance, for example, *dist*(*task_dFC*, *HE*) versus windowed RT.

To further evaluate those relationships, a linear mixed effects (LME) model was formulated on the group level by treating subject and window as random effects (Bates et al., 2014). The *p* values were computed using likelihood ratio tests, by comparing the goodness of fit of a full model and a reduced model (one with the fixed effect, e.g., *B*_*T*_, in question and one without). The *t* values were obtained from the full LME model (df = 177). The results of 2-back memory task are reported in Table 2. Eight out of nine relations were proven significant, and there was a considerable trend toward significance between *B*_*T*_ and missing rate (*p* = 0.090). These results suggest that as task performance degrades, dFCs appear more similar to the LE-FC pattern, and less similar as the task-specific HE-FC pattern. Moreover, an increased brain integration level (*B*_*T*_) was found to be associated with improved task performance. Those relations were true only for the working memory task, as no consistently significant relation was found that correlated task performance for either the math or the video. Results of math and video task can be found in Supporting Information Table S1 (Xie, Gonzalez-Castillo, Handwerker, Bandettini, Calhoun, Chen, Damaraju, Liu, & Mitra, 2018).

**Table 2.** Results for 2-back memory task

***t* value (*p* value)**	**RT**	**Response accuracy**	**Missing rate**
*dist*(*WM_dFC*, *HE*)	2.82 (0.005)	−2.47 (0.014)	3.30 (0.001)
*dist*(*WM_dFC*, *LE*)	−3.93 (< 0.001)	4.28 (< 0.001)	−3.93 (< 0.001)
*B*_*T*_	2.68 (0.027)	2.35 (0.020)	−1.72 (0.090)

### Group-Level HE/LE-FC Contrast Pattern

In this section, we focused on the difference between the HE-FC and LE-FC patterns during the 2-back task in order to pinpoint potential key FC links for engagement level. After obtaining the HE-FC and LE-FC matrices, each of which contains 61 independent components (ICs), we computed the difference between each subject’s HE-FC and LE-FC pattern, that is, FC(HE,subN) − FC(LE,subN), and performed a one-sample one-sided *t* test for each of 61 × 60/2 = 1,830 links. We retained links that were significant at an FDR-corrected *p* value of 0.01. We also assigned network labels by computing the spatial overlap between ICs and eight canonical networks from the Shen atlas (Shen, Tokoglu, Papademetris, & Constable, 2013). For seven ICs with almost equal overlap with more than one canonical network in the Shen atlas, the IC labels were manually determined by visually checking the ICs’ spatial patterns and compared against previous studies (Allen et al., 2014; Xie et al., 2017).

**Figure 3.**
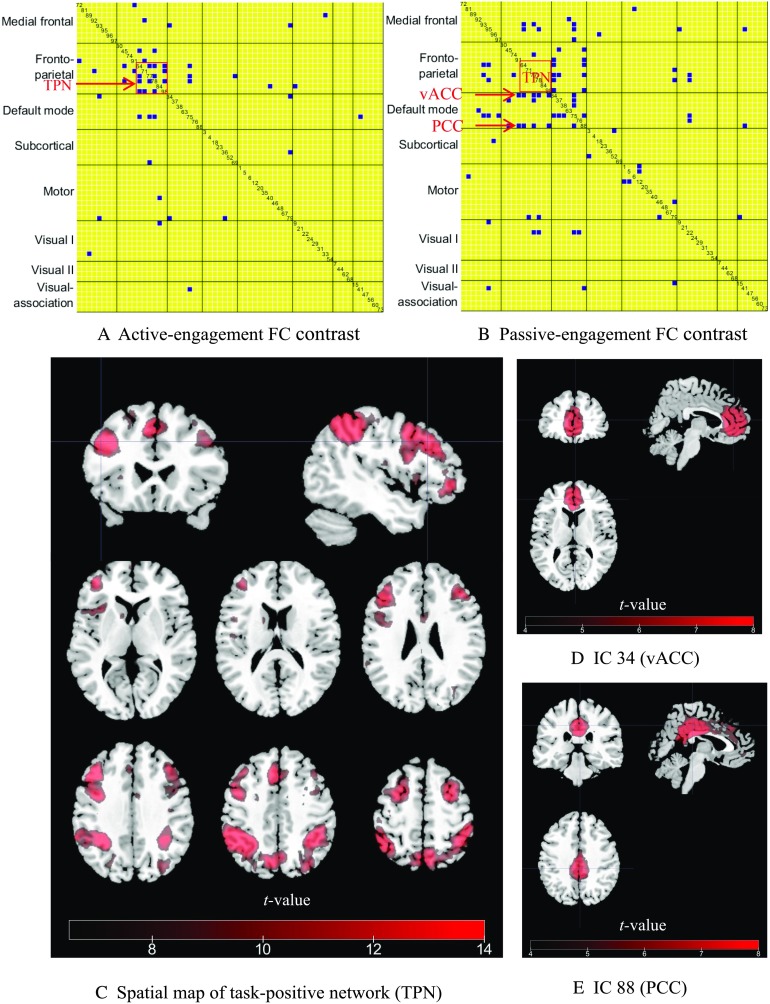
FC contrast maps between HE-FC and LE-FC during 2-back working memory task and spatial maps of ICs highlighted in two contrast maps. (A) Active-engagement FC contrast (HE > LE). Only links that were significant at a FDR-corrected *p* value of 0.01 were kept. The IC index is also displayed along the diagonal cell. The task-positive network (TPN) for working memory task (IC 64, 77, 78, 84, and 98) are highlighted by the rectangle. (B) Passive-engagement FC contrast (LE > HE). IC 34 and 88 pointed at by arrows are ventral anterior angular cortex (vACC) and PCC, respectively, which are more coupled to TPN during passive engagement. (C) A composite spatial map of task-positive ICs. (D) The spatial map of IC 34 (vACC). (E) The spatial map of IC 88 (PCC).

This yielded two group-level engagement-specific FC contrast patterns that were consistent across subjects, that is, the active-engagement (HE > LE) and passive-engagement (LE > HE) contrast as shown in Figure 3. If a link appears significant in the active-engagement contrast, it indicates a stronger link is associated with improved task performance. Conversely, a significant link in the passive-engagement contrast suggests that stronger coupling between those ICs could be detrimental to task performance. Figure 3A reveals clusters among fronto-parietal ICs with significantly increased connectivity during the active engagement of the working memory task. Figure 3B shows a higher number of connections within default mode, as well as an increased number of links between fronto-parietal and default-mode ICs during the passive engagement period.

To better interpret the results in Figure 3A and 3B, we sorted the ICs associated with the working memory task by matching the ICs’ spatial maps with the activation map generated from NeuroSynth (Yarkoni et al., 2011; http://neurosynth.org/) using the term “working memory” (see Supporting Information Figure S1, Xie et al., 2018). Five task-positive ICs were identified in this manner, namely IC 64, 77, 78, 84, and 98 as shown in Figure 3C. These ICs include portions of the inferior frontal gyrus, superior parietal lobule, dorsolateral prefrontal cortex, and inferior parietal lobule. Moreover, two default-mode ICs (i.e., IC 34 and 88) were significantly coupled with task-positive ICs during the passive engagement condition (highlighted by the arrows in Figure 3B), were identified as ventral anterior angular cortex (vACC) and posterior cingulate cortex (PCC) as shown in Figure 3D and 3E.

## DISCUSSION

In this study, we successfully replicated the relationship observed by Gonzalez-Castillo et al. (2015) between *k*-means clustering accuracy and subject overall task performance, and our results also suggest the integrity of the clustering structure of dFCs reflects individual’s task performance (Figure 2) while using a group ICA approach rather than an atlas-based approach. We extended the earlier work by further hypothesizing that short-term engagement level (i.e., the amount of effort dedicated to the task during a short period of time) may be encoded in the whole-brain dFC. We tested three engagement markers extracted from whole-brain dFC patterns, namely dissimilarity between dFCs and FC patterns representing active engagement (high-engagement FC or HE-FC); dissimilarity between dFCs and FC patterns representing passive engagement (low-engagement FC or LE-FC); and the level of brainwide integration level indexed by mean participation coefficients. Those metrics were used to relate to the short-term engagement level reflected by windowed task performance through a mixed effects model. Experimental results only partially confirmed our initial hypothesis. In particular, the expected relationship was only observed for the working memory task, but not for the math and video tasks.

### Behavioral Relevance of Whole-Brain dFC

An increasing number of studies has focused on the behavioral relevance of whole-brain dFC (Cohen, 2017). In the current study, we showed that for the memory task (see Table 2), worse task performance was associated with dFCs with lower similarity to HE-FC patterns, and higher similarity to LE-FC patterns, as well as smaller participation coefficients (less integrated brain configuration). This suggests that the degree of deviation of dFCs from the HE-FC patterns (FC pattern of active engagement) and LE-FC patterns (FC pattern of passive engagement) as well as the degree of global FC integration may indeed reflect the extent of task engagement, as originally hypothesized, although only limited to the confines of the memory task.

When focusing on the global FC pattern, we showed that higher similarity to HE-FC patterns was associated with better short-term task performance. This is in line with previous studies showing that decreased dFC variability is related to improved task performance including increased accuracy (Elton & Gao, 2015) and more stable response times (Hutchison & Morton, 2015). In other words, the fact that reduction in dFC variability associated with better task performance highlights the importance of maintaining a stable brain functional organization that is optimized for a given task (HE-FC pattern) for successful task performance. The remaining dFC variability during task performance may reflect some degree of residual mind wandering and loss of focus as suggested by Elton and Gao (2015). On the other hand, we quantified task disengagement level by using the similarity between dFC patterns and non-task-specific LE-FC (rest *k*-means centroids). Using a 64-task dataset, Cole and colleagues noted that task-evoked FC changes from rest are often similar to one another, indicating the existence of a task-general network architecture (Cole, Bassett, Power, Braver, & Petersen, 2014). Hence, failure of evoking such task-general network architecture may signal disengagement from task, and result in dFCs with higher similarity to the rest FC pattern.

When switching our attention to specific FC links and networks, careful examination revealed five frontal-parietal ICs (IC 64, 77, 78, 84, and 98) showing group-level increase during active engagement of working memory than passive engagement (Figure 3C). Using the meta-analysis tool NeuroSynth (Yarkoni et al., 2011), we observed how those task-positive ICs showed considerable overlap with the so-called canonical “working memory” regions, such as dorsolateral prefrontal cortex and superior parietal lobule (Meyer & Lieberman, 2012). Moreover, stronger connectivity was also found between those task-positive ICs and ventral anterior angular cortex and PCC, part of default-mode network, during the passive-engagement condition (Figure 3D and 3E). In other words, greater decoupling between the task-positive and default-mode network is expected during active-engagement period. This observation is in line with studies linking the strength of the anticorrelation between those two networks with cognitive performance. For example, stronger anticorrelation between task-positive and default-mode network was found to be associated with faster reaction times (Thompson et al., 2013) and less variable reaction times (Kelly et al., 2008). Wang et al. (2016) also found that reduced anticorrelation between the default-mode and attention networks was associated with more frequent eyelid closure. Although the underlying mechanism remains to be further elucidated, one explanation involves the competition between internally and externally oriented cognition (Boveroux et al., 2010).

When turning our attention to global integration levels as a way to track task engagement, we were able to successfully replicate the findings in Shine et al. (2016) using Pearson’s correlation instead of multiplication of temporal derivatives (MTD) as a connectivity index. MTD is calculated as the window-averaged dot product of first-order derivatives of two time series. Our result supports the original claim by Shine et al. (2016) that a globally integrated and coordinated brain functional topology, as reflected by higher participation coefficients, might facilitate communication among brain areas that would otherwise remain segregated during active working memory task performance. Moreover, our result evidences the robustness of the link between global network integration level and working memory cognitive performance against different dFC measures as well as parcellation schemes (group ICA in current study vs. Gordon atlas in Shine et al., 2016). It is worth noting that the relationship between task performance and brainwide integration/segregation level might depend on the underlying cognitive context. During the preparation phase of a visual discrimination task, Ekman et al. (2012) reported that an enhanced integration level among task-relevant regions and a reduced integration level within task-irrelevant areas. The difference in the cognitive context could explain the discrepancy between the conclusion of Shine et al. (2016) and Sadaghiani et al. (2015), in which Sadaghiani and collegues found that a more modular brain organization was linked with better auditory detection task performance. Given the complex nature of the working memory task, it recruits more brain regions that are widely distributed across the brain compared with those an auditory detection task would, which may lead to the opposite conclusion.

A recent study by Schultz and Cole (2016) showed that subjects with higher performance on tasks such as language, relational reasoning, and memory had smaller static FC reconfiguration between such tasks and rest. In other words, Schultz and Cole concluded that task FC patterns of good performers were more similar to their rest FC pattern than those of bad performers. It was argued by the authors that individuals with less FC reconfiguration might modify network connectivity more efficiently to achieve task goals. Although our results may seem to contradict Schultz and Cole, we focused on the dynamical aspects of FC and window-to-window performance fluctuation, whereas they studied static connectivity patterns by using complete scans and their relation to intersubject performance differences. The distance between a rest and task FC map is less relevant to our measure of behavior than the relative distance of a specific window’s FC pattern and to HE/LE-FC patterns. As such, observed discrepancies could simply be a result of the difference in temporal scale and analysis-level (intrasubject in ours vs. across-subject in theirs) across both works.

### Post Hoc Analysis on Negative Findings for Math and Video Tasks

We failed to find a significant linear relationship between windowed connectivity and behavioral metrics for the math and the visual search (video) task. We performed a series of post hoc analyses in order to better understand what might have led to such negative results. Those analyses are discussed in this section.

To evaluate our hypothesis, variability in behavioral metrics should be driven primarily by task engagement levels. Post hoc evaluation of behavioral metrics following our negative results for the math and video task suggests otherwise. In particular, our data suggests (see Figures S2 and S3 in the Supporting Information, Xie et al., 2018) that intertrial variability of our behavioral metrics were confounded by other factors, such as trial difficulty. Despite our efforts to account for overall intersubject differences in performance (i.e., adopting a LME model), these additional confounds remain present, potentially masking window-to-window task engagement levels.

The visual search task involves free viewing a natural scene (a prerecording of a live fish tank without fixation), target directed saccades (look for the emergent red crosshair), and target identification (decide whether the target is a clown fish). As such, reaction times for this task can be divided into saccade latency, target localization latency, and identification latency (Castelhano, Pollatsek, & Cave, 2008). The stimuli are perceived faster and more accurately when they are near the target of an upcoming saccade (Shepherd, M., Findlay, & Hockey, 1986), hence the saccade latencies would be heavily influenced by the foveal location prior to the appearance of the target (which is independent of task engagement), but expected to be highly variable across trials.

For the math task, subjects were asked to select the correct answer from two given options. Math trials involved addition and/or subtraction of three numbers between 1 and 10. One robust phenomenon in mathematical cognition is known as the problem size effect, which refers to the fact that solution latencies (reaction time) and error rate increase with larger operands (Ashcraft & Guillaume, 2009). Another potential confounding factor in the math task is intertrial alternations between retrieval or calculation strategies (Van Beek et al., 2014). Both of these confounds may have played an important role determining solution latencies, therefore masking task engagement levels.

On the contrary, the memory task is less prone to the confounding effects of the abovementioned factors, as in our study the number complexity (Sternberg, 1969), and strength (Froeberg, 1907) of stimuli, intertrial interval, and memory load remained constant throughout the task. Unlike the math and video tasks (Figures S2 and S3, Supporting Information, Xie et al., 2018), no relationship between individual’s RT and group-averaged RT was observed for the memory task, which supports the lack of systematic intertrial difficulty differences. In fact, working memory and attentional engagement have been comprehensively studied together in the past, and led to a long list of significant findings and theories (Awh & Jonides, 2001; Hampson, Driesen, Skudlarski, Gore, & Constable, 2006).

In summary, our initial hypothesis set was confirmed for one of the three candidate tasks. Post hoc detailed evaluation of behavioral metrics for the math and video tasks (those leading to negative results) suggests that their behavioral metrics may not cleanly reflect task engagement, impeding evaluation of our hypotheses. In the meantime, we believe the results presented here provide evidence in support of our original claim that dFC patterns may contain information that goes beyond task identification, and could be a valuable index for tracking task engagement levels.

### Limitations and Future Directions

Our study has some important limitations, most of which result from its exploratory nature. As mentioned earlier, this study used data acquired under a paradigm previously used (Gonzalez-Castillo et al., 2015; Xie et al., 2017). This was done so that results could be better interpreted within the context of those previous studies, as well as for the previously reported good separation of these tasks via dFC. With hindsight, two of the three tasks were not well suited for our goals, as their recorded behavioral metrics were not driven primarily by fluctuations in engagement level. To reliably use behavioral metrics like reaction time as a proxy for task performance requires control for confounding factors such as perceived difficulty, engagement strategies, and fixation location. Although the memory task results presented here support our initial hypothesis, it would be desirable to also show that the effects observed here are generalizable across tasks. To address this question of generality, we believe that tasks such as gradual onset continuous performance task (gradCPT; Rosenberg et al., 2016) could constitute good candidates for future studies. The gradCPT task consists of stimuli that gradually transition between images of cities and mountains, and participants are instructed to respond when they perceive city scenes (which occur randomly 90% of the time) leading to frequent behavioral measures not contaminated by any of the abovementioned factors. In addition, behavioral measurements other than reaction time or accuracy that are more directly related to engagement level, such as pupil diameter, could be helpful as complementary behavioral logs.

In addition, although *k*-means clustering together with the sliding window approach has proven to be an effective method to study FC dynamics (Allen et al., 2014; Wang et al., 2016; Xie et al., 2017), we are aware of the ongoing debate on the accuracy of dFC estimation based on sliding window (Abrol et al., 2017; Laumann et al., 2016; Liégeois et al., 2017). One criticism of window-based approach is the arbitrary choice of WL (Lindquist, Xu, Nebel, & Caffo, 2014). In our case, no optimal a priori WL exists given the different intertrial intervals for the different tasks. Excessively long WL may increase stability of dFC estimation, but may hamper our ability to analyze FC and behavioral dynamics. Too short of a WL may lead to inaccurate estimation of FC dynamics and engagement level. We believe WL = 30TRs = 45 s constitutes a plausible middle ground to accommodate both factors. It should be noted that optimal WL is recommended to be 50 s without knowledge about the true dFC timescale (Hindriks et al., 2016), and a recent study comparing various WLs identified 45 s as an optimal choice (Vergara et al., 2017). Future studies should consider alternative dFC methods, such as dynamic condition correlation (Lindquist et al., 2014), dynamic coherence (Yaesoubi et al., 2015), and sparse dictionary learning (Yaesoubi, Adali, & Calhoun, 2018). It would be interesting to evaluate our approach against different parcellation schemes as well.

## METHODS

### Subjects and Experimental Paradigm

The dataset consisted of two datasets with a total number of 25 subjects (age 26.25 ± 5.15 years, 9 men). The first dataset with 17 subjects was previously studied (Gonzalez-Castillo et al., 2015, available at https://central.xnat.org, project ID: FCStateClassif), and the second dataset with additional 8 subjects was later collected for another study by using a similar protocol. Functional runs of the subjects were obtained on a Siemens 7 Tesla MRI scanner using a 32-element receive coil (Nova Medical) with a gradient recalled, single shot, echo planar imaging (gre-EPI) sequence with TR = 1.5 s, TE = 25 ms; FA = 50°, 40 interleaved slices; FOV = 192 mm; in-plane resolution, 2 × 2 mm; slice thickness, 2 mm.

**Figure 4.**
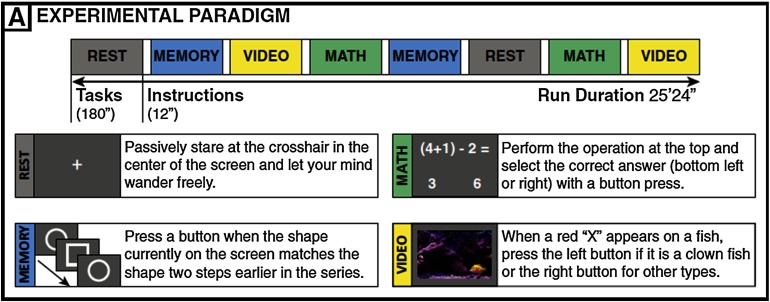
Experimental paradigm from Gonzalez-Castillo et al. (2015).

The subjects were scanned continuously for approximately 25 min (1,017 TRs) as they engaged and transitioned between four different mental tasks (math, memory, video, and rest) as shown in Figure 4. Each task block lasted for 120 TRs (180 s). There were instructions between every two task blocks for 8 TRs (12 s). Each task was repeated twice, and the order of task blocks was randomized so that each task was followed by a different task. Below is a summary of four task paradigms.

During the resting-state scan, subjects were asked to passively fixate on the crosshair in the center of the screen.

For the memory task, subjects were shown a continuous sequence of five different geometric shapes that appeared in the center of the screen every 3 s (shapes appeared on the screen for 2.6 s, followed by a blank screen for 0.4 s). Subjects were asked to press the button when the current shape matched that of two shapes before. There was a total number of 60 memory trials per block.

For the math task, the subjects were instructed to choose one correct answer among two choices for a math operation involving subtraction and addition of three numbers between 1 and 10. The operation remained on the screen for 4 s followed by a blank screen for 1 s. There was a total number of 36 math trials per block.

For the video (visual search) task, a short video clip of fish swimming in a fish tank was presented, and subjects were asked to identify whether the fish highlighted by a red crosshair is a clown fish by pressing left button (or right button if the target fish is not a clown fish). Each cue (i.e., the red crosshair) lasted for 0.2 s, and there was a total number of 16 trials per task block.

### Data Preprocessing

With a combination of toolboxes (SPM and AFNI) and customized code developed by the Mind Research Network, the imaging data underwent the following preprocessing steps: removal of the first four volumes; slice timing correction using middle slice as the reference slice; motion correction; despiking (*3dDespike*) to mitigate the impact of outliers; detrending (*3dDetrend* up to eighth order given the relatively long scan time); spatial normalization to Montreal Neurological Institute space; spatial smoothing with a Gaussian kernel with FWHM = 4 mm; and, finally, intensity normalization to percentage signal change.

### Postprocessing and Functional Network Connectivity Estimation

The group ICA was performed using the GIFT toolbox (http://mialab.mrn.org/software/gift/) with the model order (number of components) equal to 100. Principal components analysis was adopted to retain 120 principal components (PC) at the single-subject level and the expectation maximization algorithm was applied to retain 100 PCs at the group level. The Infomax ICA algorithm was repeated 20 times using ICASSO (http://www.cis.hut.fi/projects/ica/icasso) with random initialization, and aggregate spatial maps were estimated as the modes of the component clusters. Subject-specific time courses and spatial maps were estimated using the GICA1 back reconstruction method (Erhardt et al., 2011). A subset of 61 ICs were manually identified for further analysis based on the expectations that ICs should exhibit peak activations in gray matter, and should have time courses dominated by low-frequency or task-frequency fluctuations (Allen et al., 2014). Time courses underwent motion-related variance regression (motion parameters and the first derivatives), and then were band pass filtered with a sixth-order Butterworth band-pass filter (0.0222–0.18 Hz). The upper cut-off frequency was chosen to be 0.18 Hz to avoid confounds arising from task motor responses, for example, one button press was required every 5 s during math task. The low cut-off frequency was set according to the WL (45 s) to remove spurious fluctuations as suggested by Leonardi and Van De Ville (2015). The time courses were shifted by 6 s or 4 TRs to approximately account for hemodynamic delay and segmented with nonoverlapping windows with WL (45 s) yielding a total number of 32-windowed time series (8 for each task) for each subject. On average, each window contains 15 trials for working memory task, 9 for math task, and 4 for visual search task. The dFCs were calculated as Pearson’s correlation of those windowed time series, on which Fisher transformation was then applied. One subject (SBJ 15) was dropped because of high similarity across all dFCs.

### *K*-Means Clustering

*K*-means clustering was applied on the dFCs as an unsupervised vector quantization tool to explore the intrinsic structures of FC dynamics for each individual. The number of clusters was set to four, and Pearson’s correlation was used as a distance measure. The maximum number of iterations for the *k*-means algorithm was set to 1,000 to ensure the algorithm converges, and *k*-means ++ (Arthur & Vassilvitskii, 2007) was used to choose initial cluster centroid positions. The distance-to-centroid was recorded after convergence and later correlated with windowed behavioral measures.

### Window-by-Window Participation Coefficients

This part of analysis was performed to validate the findings in (Shine et al., 2016), which used a slightly different connectivity measure rather than Pearson’s correlation coefficients. The whole analysis was carried out using brain connectivity toolbox (Rubinov & Sporns, 2010; https://sites.google.com/site/bctnet/).

First, the Louvain modularity algorithm for community detection was employed to estimate time-varying community structure by iteratively maximizing the modularity statistics. The goal is to further parcellate the ICs into communities so that the within-module connectivity is stronger than the between-module connectivity. The Louvain modularity (Blondel, Guillaume, Lambiotte, & Lefebvre, 2008) was repeated 500 times for each dFC and a consensus partition was achieved with the consensus clustering algorithm introduced in (Lancichinetti & Fortunato, 2012). Then, the participation coefficient (*B*_*T*_), which measures the between-module connectivity strength, was computed for each window using Equation 1:

$$B_{iT}=1-\sum_{s=1}^{N}{\left(\frac{\kappa_{isT}}{\kappa_{iT}}\right)}^{2}$$ (1)

where *B*_*iT*_ is the participation coefficient of IC *i* for window *T*; *κ*_*isT*_ is the strength of the positive connections of IC *i* belonging to the module *s* for window *T*; *κ*_*iT*_ is the total strength of all positive connections of IC *i* for window *T*; and *N* is the total number of modules detected with the Louvain modularity algorithm. Hence, the participation coefficient is between zero (all links are within its own module) and one (links are uniformly distributed among all the modules), as detailed by Guimera and Amaral (2005).

Finally, the participation coefficients were averaged across all the ICs of each window to achieve mean window-by-window mean participation coefficients (*B*_*T*_), which represented the level of global integration for window *T* and were later correlated with average behavioral measures for that window.

### Group-Level Linear Mixed Effects Analysis

LME analysis has been used to conduct group analysis for fMRI studies, as this method can address issues such as repeated measurements per person, missing data, and multiple subject-grouping, and therefore leads to increased statistical power as well as controls for within-individual variation (Beckmann, Jenkinson, & Smith, 2003; Chen, Saad, Britton, Pine, & Cox, 2013).

For this study, we used R (R 3.4.2) and lme4 package (Bates et al., 2014) to perform the LME analysis. The behavioral metrics (e.g., windowed RT) were expressed as a linear combination of a set of independent variables. The fixed effects were metrics derived from whole-brain windowed FC matrices, for example, distance-to-centroid or windowed mean participation coefficient. The random effects included intercepts for subjects (within-subject variation) and windows (within-window variation as all subjects go through the same experimental paradigm). All variables were *z* scored before the LME analysis. The *p* values were computed by likelihood ratio tests, that is, comparing the full model with the fixed effect in question against a reduced model without the fixed effect, to determine which model better fits the data. The *t* values were obtained from the full LME model. Moreover, framewise displacement (FD; Power, Barnes, Snyder, Schlaggar, & Petersen, 2012) was used as a proxy for head motion, and windowed FD was included in both models as a fixed effect to rule out the possibility that effect was merely driven by head motion.

### Group-Level Contrast Pattern

We compared the group-level FC patterns associated with high-engagement (HE) and low-engagement (LE) level by using a one-sample *t* test. More specifically, the difference between *k*-means centroid labeled as memory and rest was computed on the subject level, yielding a 61 × 61 matrix and 61 × 60/2 = 1,830 FC links for each subject. We performed a one-sample one-tailed *t* test on a given link of all 24 subjects, followed by FDR to correct for multiple comparison, and we only kept the links with FDR-corrected *p* value smaller than 0.01. Two group-level contrast patterns were generated, namely active-engagement (HE > LE) contrast and passive-engagement (LE > HE) contrast. A significant link in active-engagement contrast indicates the strength of the link is stronger for the HE condition. A significant link in passive-engagement contrast means the coupling between the two nodes is stronger during the LE condition, hence weaker coupling (or stronger decoupling) is to be expected during the HE condition.

The network labels were assigned by computing the spatial overlap between IC’s spatial maps and the spatial masks of eight canonical networks from the Shen atlas (Finn et al., 2015; Shen, Tokoglua, Papademetrisa, & Constable, 2013), and the one network with maximum overlap was chosen. For seven ICs that were shared equally by more than one network, the network assignment was determined by visual inspection and comparison with previous studies (Allen et al., 2014; Xie et al., 2017).

To identify the task-positive ICs, the activation pattern of working memory was obtained from NeuroSynth (Yarkoni et al., 2011; http://neurosynth.org/) using the keyword “working memory.” Neurosynth exported a whole-brain *z*-score map representing the likelihood that a voxel being activated associated with working memory from 901 studies. The working memory activation pattern generated by NeuroSynth can be found in Supporting Information Figure S1 (Xie et al., 2018).

## AUTHOR CONTRIBUTIONS

Hua Xie: Conceptualization; Formal analysis; Investigation; Methodology; Software; Writing – original draft; Writing – review & editing. Javier Gonzalez-Castillo: Conceptualization; Data curation; Formal analysis; Investigation; Methodology; Resources; Supervision; Writing – original draft; Writing – review & editing. Daniel A. Handwerker: Data curation; Supervision; Writing – review & editing. Peter A. Bandettini: Funding acquisition; Supervision; Writing – review & editing. Vince D. Calhoun: Funding acquisition; Supervision; Writing – review & editing. Gang Chen: Formal analysis; Writing – review & editing. Eswar Damaraju: Formal analysis. Xiangyu Liu: Writing – review & editing. Sunanda Mitra: Funding acquisition; Supervision; Writing – review & editing.

## FUNDING INFORMATION

Peter A. Bandettini, National Institute of Mental Health (http://dx.doi.org/10.13039/100000025), Award ID: ZIAMH002783. Vince D. Calhoun, National Institute of Mental Health (http://dx.doi.org/10.13039/100000025), Award ID: R01EB020407. Vince D. Calhoun, National Institute of General Medical Sciences (http://dx.doi.org/10.13039/100000057), Award ID: P20GM103472. Vince D. Calhoun, National Science Foundation (US), Award ID: 1539067. Portions of this study used the high-performance computational capabilities of the HPC Biowulf Cluster at the National Institutes of Health, Bethesda, MD (http://hpc.nih.gov).

## TECHNICAL TERMS

Functional connectivity: Functional connectivity refers to statistical dependency between signals from spatially distributed brain regions.
Spatial independent component analysis: Spatial ICA decomposes four-dimensional fMRI data into spatially independent maps and time courses.
*K*-means clustering: *K*-means clustering is an unsupervised clustering method with the aim to partition data into *k* clusters so that each observation belongs to the cluster with the highest similarity, and the cluster centroid serves as a prototype of the cluster.
Task-evoked functional connectivity dynamics: Temporal evolution of functional connectivity reconfiguration during task performance.
Brain segregation/integration: Connections occur primarily within or across functional networks.
Linear mixed effect analysis: Linear mixed effects analysis is extension of linear regression models for data that are collected in groups that has both random and fixed effects.
Likelihood ratio test: A likelihood ratio test is a statistical test used for comparing the goodness of fit of two models (full vs. reduced model).
