# Supplementary material for: Time-varying whole-brain functional network connectivity coupled to task engagement
Source: Netw Neurosci. 2018 Oct 1;3(1):49–66. doi: 10.1162/netn_a_00051 (PMC6326730; doi:10.1162/netn_a_00051)
Supplement: Supplementary file 1 [file netn-03-49-s001.pdf]

Xie, H., Gonzalez-Castillo, J., Handwerker, D. A., Bandettini, P. A. Calhoun, V. D., Chen, G., … & Mitra, S. (2018). Supporting Information for “Time-varying whole-brain functional network connectivity coupled to task engagement." *Network Neuroscience*, *3(1),* 49–66. https://doi.org/10.1162/netn_a_00051

Time-varying Whole-brain Functional Network Connectivity Coupled to Task Engagement (Supplemental Material)

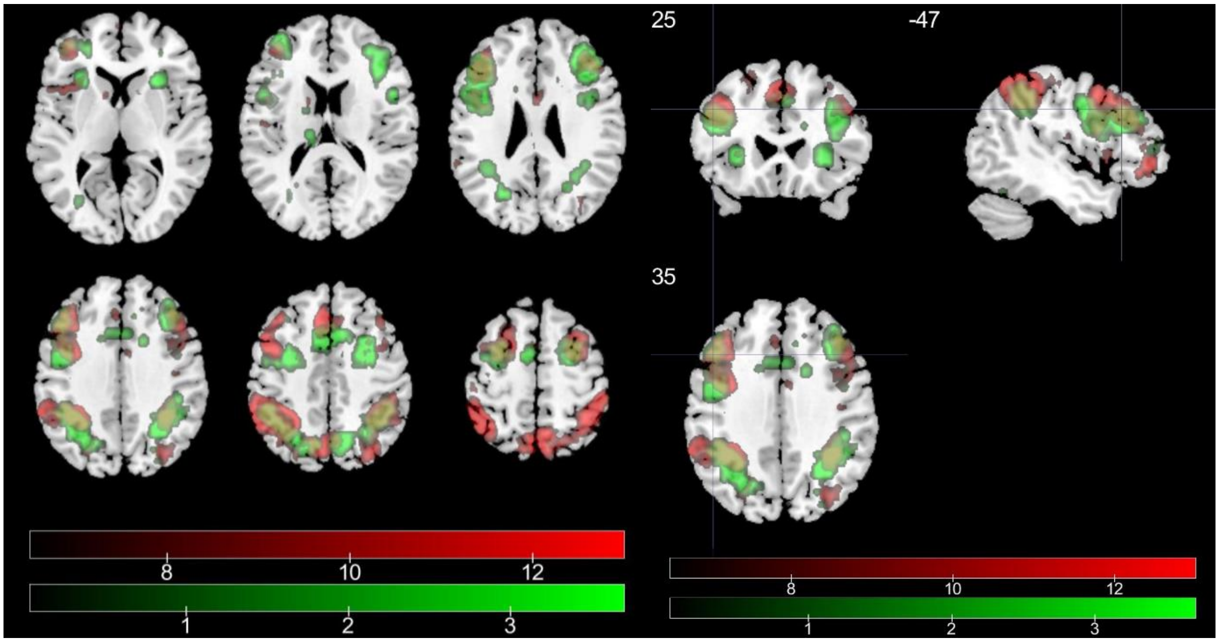

Fig S1. Activation probability map (green, z-score) from Neurosynth generated by using the term “working memory”, compared against the task-positive networks identified from ICs (red, t-value). There is considerable overlapping in regions associated with working memory function such as inferior frontal gyrus, superior parietal lobule, dorsolateral prefrontal cortex, and inferior parietal lobule.

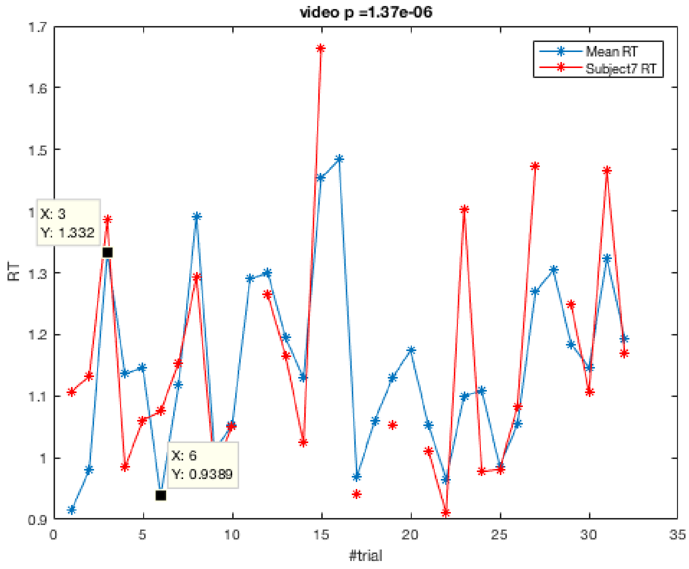

(a)

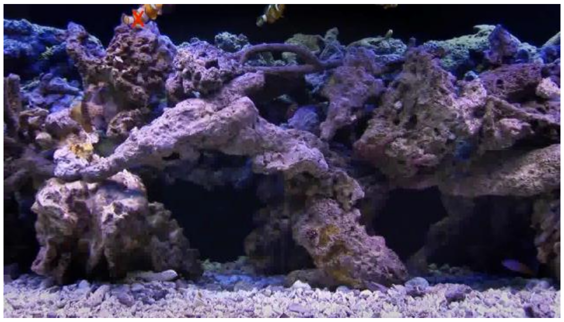

(b)

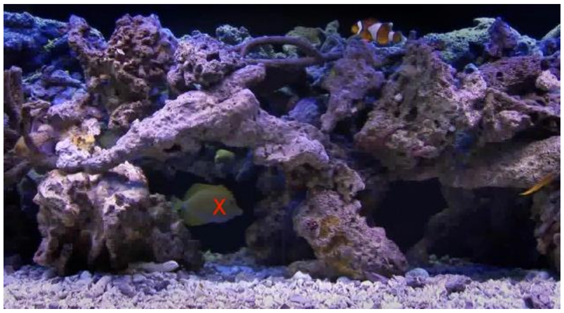

(c)

Fig. S2. An example of subject's RT correlates with group-averaged RT during the video task, indicating inter-trial difficulty difference. (a) RT of subject 7 correlated with group-averaged RT during the video task. X-axis: trial number, Y-axis: RT. (b) Cue of trial 3. (c) Cue of trial 6.

It can be observed from Fig. S2 that cue of trial 3 is on the boundary of the screen while cue of trial 6 is relatively in the center, which could be one of many reasons that caused longer RT for trial 3. Other potential factors could be contrast, size of the target fish, and transience of the target (how fast is the target fish when the cue appears). It is not surprising to observe such effect (subject's RT correlated with group-averaged RT) among 20 out of 24 subjects after FDR correction.

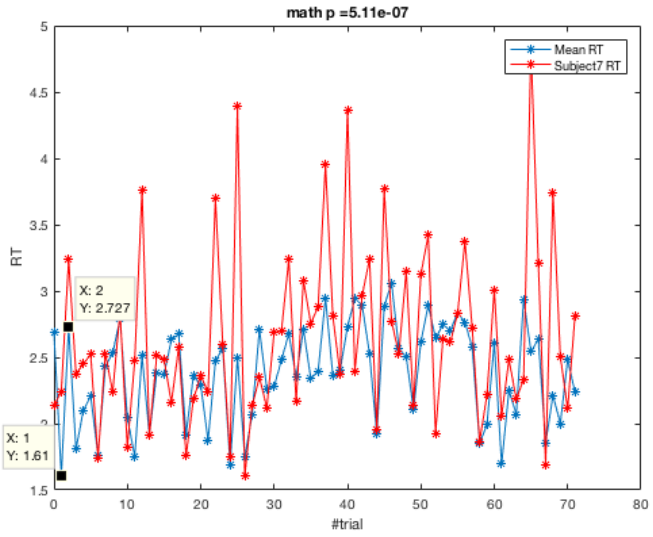

(a)

(b) TRIAL 1 [(6 + 1) + 1 = | 4,8]

(c) TRIAL 2 [(7 - 5) - 4 = | -2,-7]

Fig. S3. An example of subject's RT correlates with group-averaged RT during the math task, indicating inter-trial difficulty difference. (a) RT of subject 7 correlated with group-averaged RT during the math task. X-axis: trial number, Y-axis: RT. (b) Math question of trial 1. (c) Math question of trial 2.

Math task shares the same issue that behavior measures were confounded by the trial difficulty as shown in Fig S3. Subjects were all shown the exact same questions for the same trial (except for subject 3). It is natural to see that on average subjects spend more time on trial 2 than trial 1, as the first trial is barely a single-digit addition which does not require much mental computation, while the second trial involves negative number therefore taking more time. Trial difficulty may vary also depending whether it involves carry operation (summation over 10). RT of 23 subjects significantly correlated with group-averaged RT after FDR correction.

However, we did not observe such effect for the 2-back memory task. The common way to alter trial difficulty in working memory task usually involves degradation of the stimuli [1], change of memory load (one, two, or three items) and temporal demand (interstimulus intervals of two, three, or four) [2], none of which were applied in our study (five items and constant interstimulus intervals).

Hence, we concluded that the behavior measures during math and video task were confounded by the trial difficulty and were no longer a suitable proxy of the task engagement level.

Table S1. Results of video and math task against reaction time (RT).

t-value (p-value)	Video.RT	Math.RT
dist(dFC,HE)	0.14 (0.889)	-1.24 (0.215)
dist(dFC,LE)	-0.10 (0.920)	2.339 (0.022)
B_T	-0.737 (0.461)	-1.56 (0.119)

As shown in Table S1, no consistent relationship can be observed between engagement metrics derived from whole-brain dFC and RT. We only showed results for RT given the high correlation among three behavioral measurements.
